# Different Brain Regions Support Deliberation during Food Choice in Disordered and Healthy Eating

**DOI:** 10.1523/JNEUROSCI.0441-26.2026

**Published:** 2026-06-16

**Authors:** Alexandra F. Muratore, Eileen A. Hartnett, Karin Foerde, Blair W. Uniacke, B. Timothy Walsh, Joanna E. Steinglass, Daphna Shohamy, Akram Bakkour

**Affiliations:** ^1^New York State Psychiatric Institute and Department of Psychiatry, Columbia University Irving Medical Center, New York, New York 10032; ^2^Department of Psychology and Mortimer B. Zuckerman Mind Brain Behavior Institute, Columbia University, New York, New York 10027; ^3^Department of Psychology, University of Amsterdam, Amsterdam 1018 WS, The Netherlands; ^4^The Kavli Institute for Brain Science, Columbia University, New York, New York 10027; ^5^Department of Psychology, Institute for Mind and Biology, and Neuroscience Institute, University of Chicago, Chicago, Illinois 60637

**Keywords:** anorexia nervosa, computational psychiatry, decision-making, fMRI, food choice, restrictive eating

## Abstract

The brain is wired to drive behavior toward foods that are high in sugar and fat. Yet, individuals with anorexia nervosa (AN) prefer low-sugar and low-fat foods to the point of starvation and even death. Here, we aimed to understand how alterations in decision-making processes and associated neural activity contribute to the pattern of maladaptive food-related decisions observed in AN. We combined decision-making tasks with computational modeling of behavior and fMRI to examine food-specific and general (nonfood)-related decisions in female individuals with AN and healthy controls (HC). Results from this preregistered study suggest that patients with AN, like HC, employ a decision-making process that relies on sampling and evaluating evidence, regardless of the type of decision. However, neural activity patterns differed between groups when deliberating about what to eat: food choice-related activation among HC was localized to the hippocampus, whereas individuals with AN engaged both the hippocampus and the striatum, regions apparently serving as sources of evidence in the decision process. These findings suggest that the maladaptive reversal of food preferences in AN may be attributable to reliance on different inputs to the process that leads to restrictive food choice rather than a maladaptive decision process per se.

## Significance Statement

Disordered eating often involves maladaptive choices about what to eat. The present research shows that the decision-making process unfolds similarly in healthy individuals and patients with anorexia nervosa but the brain regions that support deliberation about what to eat differ. Together, the findings suggest that the inputs to the decision process rather than the decision process per se should be a point of focus in the search for novel treatments of eating disorders.

## Introduction

Many maladaptive behaviors reflect excessive pursuit of reward. The eating disorder anorexia nervosa (AN), however, presents the opposite pattern: individuals with AN persistently reject highly palatable foods. In contrast to the natural draw to calorie-dense foods rich in sugar and fat preserved across species (Drewnowski and Almiron-Roig, 2010), patients with AN preferentially choose low-calorie foods, often to the point of starvation and even death (Steinglass et al., 2015). Understanding how such decisions arise may reveal how neural systems that normally promote reward-seeking can support the persistent avoidance of natural rewards.

The restrictive choices observed in AN are commonly attributed to altered valuation, whereby individuals with AN assign lower value to high-fat foods (Schebendach et al., 2011). However, focusing on valuation alone makes it difficult to determine whether underlying decision-making mechanisms are also altered. Maladaptive food choices may arise solely from differences in the valuation of foods (i.e., inputs) or may be attributed to differences in both the value of food and the way in which this information is transformed into action (i.e., decision process). In other words, once valuation differences are accounted for, do individuals with AN additionally differ in the cognitive and neural mechanisms by which evidence is accumulated and food-related decisions are reached?

Disentangling valuation from other aspects of decision-making is particularly important given existing evidence on the neurobiology of decision-making in AN. Previous studies have reported increased dorsal striatal activity in individuals with AN during food-related decisions (Foerde et al., 2015), which may decrease with treatment as high-fat food choices increase (Foerde et al., 2021), as well as altered functional connectivity between the dorsal striatum and dorsolateral prefrontal cortex (dlPFC) relative to healthy controls (HC). Thus, differences in food choices among individuals with AN are known and appear to be associated with neural differences, but which components of the decision process are altered remains unclear.

Sequential sampling models provide a useful framework for examining decision mechanisms. These models posit that individuals accumulate evidence for competing options until a decision threshold is reached. The drift-diffusion model (DDM) is one such model that captures both choice outcomes and reaction times (RTs), allowing researchers to infer latent components of the decision process (Ratcliff, 2002; Gold and Shadlen, 2007; Milosavljevic et al., 2010). Applying this framework to food choice therefore provides a means of testing whether individuals with AN transform decision inputs into action using the same underlying process as HC.

Importantly, food decisions rely on internal evidence, in contrast to the visuoperceptual tasks often used to study the decision process, which rely on external sensory evidence. These differences may have consequences for the neural mechanisms supporting evidence accumulation (Bakkour et al., 2019). Accordingly, in addition to comparing food-related decisions in individuals with AN and HC, we contrasted the process of food decision-making with a visuoperceptual decision-making task which removed effects of subjective value on performance. This design allowed us to test whether differences in components of the decision process are specific to decisions that rely on internally generated, value-based evidence or reflect domain-general (nonfood) alterations in decision-making. We further combined the DDM with fMRI to test whether individuals with AN engage different neural circuits when making food versus perceptual decisions.

We predicted that individuals with AN and HC would differ in how they make food-based, but not perceptual, decisions. We further predicted that the groups would differ in patterns of BOLD responses during food-related but not perceptual decision-making. Based on prior work (Foerde et al., 2015; Bakkour et al., 2019), we expected that RT during food decisions would correlate positively with striatal activity in individuals with AN and with hippocampal activity in HC, reflecting differences in the neural sources of evidence used to resolve food choices. Better understanding of how individuals with AN value food and make decisions about what to eat may provide a new perspective on how the brain makes reward-based decisions and of how this process goes awry in disease.

## Materials and Methods

Study design and hypotheses were preregistered using the Open Science Framework (https://osf.io/jh3sd).

### Participants

All participants were right-handed females, ages 16–40 years, who had normal or corrected-to-normal vision, and were competent to provide informed consent or assent. Participants were excluded if they had a history of learning disability, concussion, seizure disorder, or other neurological disorder; were pregnant; were taking medication known to have acute effects on appetite (e.g., stimulants); or had contraindications to MRI. Patients with AN were receiving inpatient treatment at the New York State Psychiatric Institute (NYSPI) and were eligible to participate if they had a DSM-5 (American Psychiatric Association, 2013) diagnosis of AN or atypical AN confirmed by the Eating Disorders Assessment (EDA-5; Sysko et al., 2015), were at a body mass index (BMI) of at least 14.5 kg/m^2^, and were medically stable. Comorbid diagnoses were assessed via Structured Clinical Interview for DSM-5 (SCID; First et al., 2015). Patients were excluded if they were experiencing acute suicidality or had a comorbid psychiatric diagnosis requiring specialized treatment. Due to high prevalence of mood and anxiety disorders among inpatients with AN, these comorbidities were not exclusionary. HC were recruited through flyers posted around Columbia University's campuses and surrounding areas in New York City. HC were eligible to participate if they had a BMI between 18.5 and 25 kg/m^2^; had no current or past psychiatric diagnoses, as assessed by the EDA-5 and the SCID; and were not on psychotropic medications.

Thirty-one patients with AN and 31 HC were recruited for participation in the study. This sample size was less than our preregistered plan to test 45 participants per group because of the impact of the COVID-19 pandemic, which led a prolonged pause in data collection and significantly impaired our ability to recruit participants even after data collection resumed. Three participants were excluded from analysis due to excessive motion (*n* = 1 HC), insufficient data (*n* = 1 HC), and substance use during treatment (*n* = 1 patient with AN).

All study procedures were approved by the NYSPI and Columbia University Institutional Review Boards. Adults provided written informed consent and adolescents provided assent with parental consent prior to participation.

### Procedures

Patients with AN took part in the study on average 2.52 ± 2.14 weeks after hospital admission. All participants had a standardized breakfast before completing a food choice task and a perceptual choice task during MRI scanning; the order of these tasks was counterbalanced across participants. The procedures are described briefly below and have been detailed extensively in a previous publication (Bakkour et al., 2019). In addition, eye tracking data were collected during all tasks. Estimated IQ was assessed using the Wechsler Abbreviated Scale of Intelligence, second edition (Wechsler, 2011). Participants also completed psychological assessments that included the Eating Disorder Examination Questionnaire (Fairburn and Beglin, 2008), the Three-Factor Eating Questionnaire (TFEQ; Stunkard and Messick, 1985), the State-Trait Anxiety Inventory (STAI; Spielberger, 1970), and the Beck Depression Inventory (BDI; Beck et al., 1961).

#### Experimental tasks

**Figure 1.**
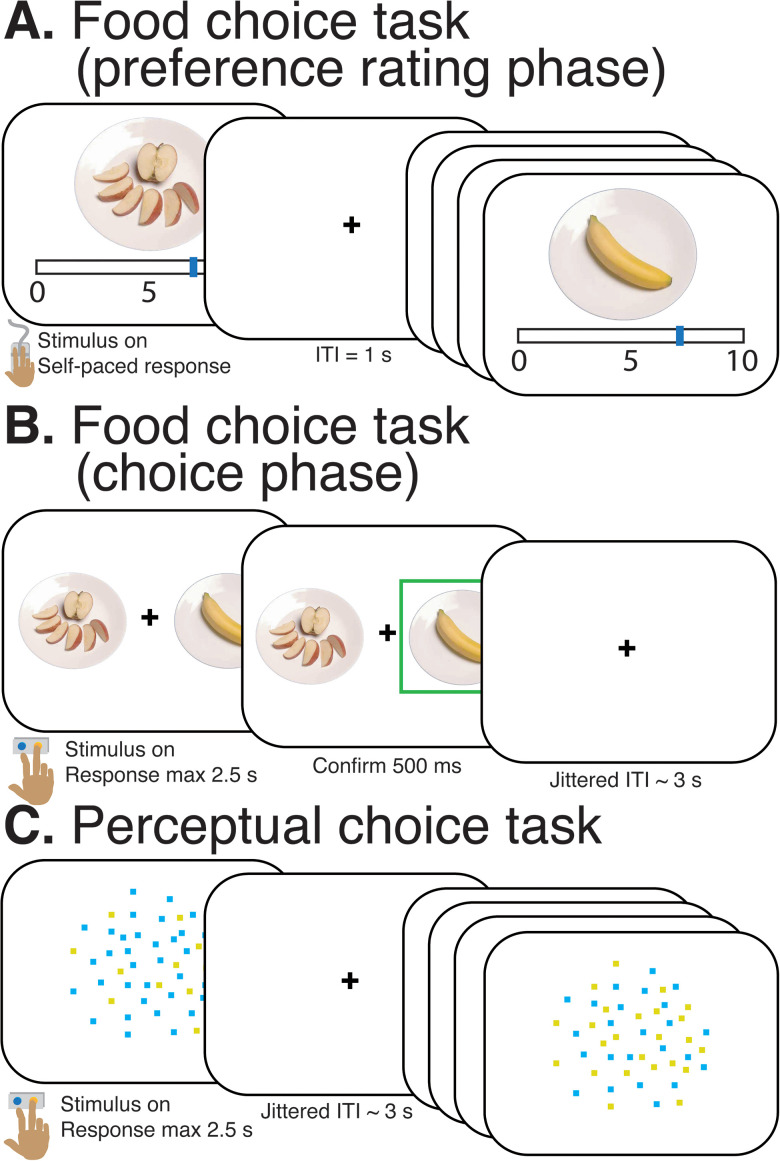
Task procedure. ***A***, Participants provided preference ratings for 60 foods that ranged from low- to high-calorie foods on a scale from 0 (least preferred) to 10 (most preferred). Participants were asked to consider a snack portion of the depicted food and rate how much they would prefer to eat that food. ***B***, During the food choice task, participants were instructed to decide which of a pair of food items they preferred on each trial. ***C***, During the perceptual choice task, participants were instructed to decide whether there were more blue or yellow dots in a dynamic display of flickering dots.

*Food choice task* (Fig. 1*A*,*B*). The food choice task consisted of a series of images of food items presented during a preference rating phase and choice phase. Stimuli were taken from the Food Folio by Columbia Center for Eating Disorders stimulus set, a publicly available database of high-resolution color photographs of food items with corresponding nutritional content (Lloyd et al., 2020). The preference rating phase was conducted outside the scanner prior to the start of the scan. In this phase, participants rated their preferences for 60 different food items from 0 (least prefer to eat) to 10 (most prefer to eat) on a visual analog scale. These ratings were *z*-scored across ratings for each participant. These *z*-scored preference ratings were then used as participant-specific estimates of subjective item value (“value”) in the subsequent choice task and fMRI analyses. Unique pairs of foods were then formed such that their difference in *z*-scored preferences (ΔValue = Rating_Item_on_right_z_ − Rating_Item_on_left_z_) varied from pair to pair. In the choice phase, participants were presented with a series of decision trials, in which the individually determined pairs of food items were presented in a random order, one pair at a time, with each food item on either side of a fixation cross. On each trial, participants were instructed to select which item they preferred by pressing one of two buttons on an MRI-compatible button-box. Participants were given up to 3 s to make a response. Once a response was made, the selected food item was highlighted for 500 ms. If no selection was made within the allotted time, the trial would end and the message “Please respond faster” would appear on the screen for 500 ms. Trials were separated by a jittered inter-trial interval (ITI) ranging from 1 to 12 s and averaged 3 s (same as for the perceptual choice task described below). Participants were presented with a total of 210 trials across three runs (70 trials each, each 7 min). Prior to the task, participants were told that they would receive a snack consisting of their chosen food item from a single, randomly selected trial to increase the likelihood that their choices would reflect true preferences. Patients were informed that, like all meals and snacks provided during treatment, the snack counted toward patient privileges. The snack item was provided after the scan.

*Perceptual choice task* (Fig. 1*C*). During the perceptual choice task, participants viewed a dynamic random dot display consisting of yellow and blue dots. Dots were presented at random locations within a central circular aperture (5 cm diameter). The proportion of blue to yellow dots was governed by color coherence, defined as the log odds that a dot is blue. Color coherence varied from trial to trial. Positive values of color coherence governed that more blue than yellow dots were present in the dynamic stimulus, while negative values of color coherence governed that more yellow than blue dots were in the stimulus. Additional details of this task can be found in a previous publication (Bakkour et al., 2019). Participants were instructed to indicate, as quickly and accurately as possible, whether there were more yellow or more blue dots in the display by selecting one of two buttons on an MRI-compatible button-box. They were given up to 2.5 s to make a response. Once a response was made, the dots display disappeared and a central fixation cross reappeared. Trials were separated by a jittered ITI ranging from 1 to 12 s and averaged 3 s. Participants were first trained on the task with feedback outside the scanner until they reached an accuracy criterion of 80% or higher over the last four blocks of 10 trials. In the scanner, participants were presented with a total of 210 trials across three runs (70 trials each, each 6.5 min) and were not provided any feedback.

#### fMRI acquisition

Imaging data were acquired on a 3 T Siemens PRISMA MRI scanner with a 64-channel head coil. High-resolution structural images were acquired using a T1-weighted (T1w) magnetization-prepared rapid acquisition gradient-echo three-dimensional sequence [repetition time (TR), 2.3 s; echo time (TE), 2.2 ms; flip angle (FA), 8°; field of view (FoV), 192 mm; matrix, 96 × 96]. Functional data were acquired using a T2*-weighted echo planar imaging sequence (slice thickness, 2 mm; TR, 1.5 s; TE, 30 ms; FA, 68°; FoV, 192 mm; matrix, 96 × 96). Oblique axial slices aligned with the anterior commissure–posterior commissure line were acquired in an interleaved fashion. Each food choice task run consisted of 284 volumes, and each perceptual choice task run consisted of 264 volumes.

### Behavioral analyses

#### Fat content

To ensure that the food choice task employed in this study captures the primary behavioral marker of AN (i.e., choice of low-fat and avoidance of high-fat foods), we calculated the difference in fat content between two foods in a pair on each trial (ΔFat = Fat_content_Item_on_right_ − Fat_content_Item_on_left_). The fat content of foods included in this study, along with a host of other nutritional information, was estimated by trained nutritionists and is published in the publicly available Food Folio by Columbia Center for Eating Disorders stimulus set (Lloyd et al., 2020). We tested whether individuals with AN in this study chose the item on the left more often when ΔFat was high when compared with HC.

#### Choice and RT

For both tasks, choices and RTs were analyzed using repeated-measures logistic and linear mixed-effects regression models, respectively. In all models, participant was included as a random effect with a random intercept. For the perceptual choice task, choices were coded as 1 if participants chose blue or 0 if participants chose yellow. For the food choice task, choices were coded as 1 if participants chose the food item on the right side of the screen or 0 if participants chose the item on the left side of the screen. Binary perceptual choice data were entered into a repeated-measures logistic regression model to calculate the odds of selecting blue in the dots choice task based on task difficulty (color coherence) and group (HC vs AN). RT data were entered into a linear regression model to determine the relationship between RT, dots choice task difficulty (|color coherence|), and group. Binary food choice data were entered into a repeated-measures logistic regression model to calculate the odds of selecting the item on the right side of the screen in the food choice task based on difficulty (ΔValue), average value of food items in a pair, and group (HC vs AN). RT data were entered into a linear regression model to determine the relationship between RT, food choice task difficulty (|ΔValue|), average value of food items in a pair, and group. Of note, we included average value of food items as a regressor to account for any differences in mean rating across items in a pair. Inclusion of this regressor in both models was a deviation from the preregistration, but the pattern of results and their interpretation did not differ from those of the preregistered behavioral regression results.

#### DDM

To determine whether there are differences in the way participants in the two groups utilize evidence in service of decision-making, we fit choice and RT data simultaneously using a DDM (Gold and Shadlen, 2007; Rangel et al., 2008; Ratcliff and McKoon, 2008; Rangel and Clithero, 2014; Ratcliff et al., 2016; Shadlen and Shohamy, 2016). Briefly, the DDM assumes that when making a decision, participants sample evidence (from the external environment in the case of the dots task, from internal sources in the case of the food choice task) and integrate these samples of evidence over time until they reach one of two decision thresholds (blue or yellow for the dots choice task, right or left item for the food choice task). Once a threshold (or bound) is reached, the participant commits to a decision. Our model extended the basic DDM and allowed the decision threshold to vary over time (i.e., the bounds remained flat for a period then decreased over time, following an exponential function), allowed for a bias in the rate of evidence accumulation for one response versus the other, and allowed for the possibility of a nonlinear monotonic relationship between the drift rate and stimulus strength (color coherence for the dots task and ΔValue for the food choice task). The details of the model and fitting procedure used here are detailed in a prior publication (Bakkour et al., 2019). The model had eight free parameters:

1. The drift rate (*v*) governed the rate of evidence accumulation.
2. The initial bound height at the start of the decision (*B*_0_).
3. The delay before the bound starts to decrease/collapse (*B*_del_).
4. The coefficient of the exponential function that governs the shape of the decreasing/collapsing bound (*B*_2_).
5. The nondecision time related to perceptual and motor processing and not related to the decision process itself (*t*_nd_).
6. The standard deviation of *t*_nd_ (*σ*_tnd_).
7. A bias to the drift rate to correct the potential that the distribution of choices and RTs for left button presses is different than that for right button presses (*μ*_0_).
8. The coefficient for a power law function applied to stimulus strength that allows for a nonlinear relationship between *v* and stimulus strength (Plaw).

These eight free parameters were first fit to data for all participants in each group and task separately for plotting purposes. We also fit the eight parameters for each participant's data for each task separately for comparison between groups. In all cases, the model was fit using the maximum likelihood estimation. This model was previously validated, and the parameters were found to be recoverable (Bakkour et al., 2019). Individual participant parameter estimates were compared across groups using the Bayesian estimation supersedes the *t* test (BEST) method (Kruschke, 2013). BEST provided a posterior distribution for group mean comparisons for each of the three main parameters of interest: *v*, *B*_0_, and *t*_nd_. We interpreted an effect as credible if the 94% highest density interval (HDI) did not include 0.

### Imaging analyses

#### Preprocessing

No preprocessing pipeline was specified in the preregistration. Results included in this manuscript come from preprocessing performed using *fMRIPrep* 20.2.6 (Esteban et al., 2017, 2018); RRID:SCR_016216, which is based on *Nipype* 1.7.0 (Gorgolewski et al., 2011); and RRID:SCR_002502. Preprocessed data were smoothed using a 4 mm smoothing kernel full-width at half-maximum.

*Anatomical data preprocessing.* One T1w image was corrected for intensity nonuniformity (INU) with N4BiasFieldCorrection (Tustison et al., 2010), distributed with ANTs 2.3.3 (Avants et al., 2008; RRID:SCR_004757). The T1w reference was then skull-stripped with a *Nipype* implementation of the antsBrainExtraction.sh workflow (from ANTs), using OASIS30ANTs as target template. Brain tissue segmentation of cerebrospinal fluid (CSF), white matter (WM), and gray matter (GM) was performed on the brain-extracted T1w using fast (FSL 5.0.9, RRID:SCR_002823; Zhang et al., 2001). A T1w reference map was computed after registration of two T1w images (after INU correction) using mri_robust_template (FreeSurfer 6.0.1; Reuter et al., 2010). Brain surfaces were reconstructed using recon-all (FreeSurfer 6.0.1, RRID:SCR_001847; Dale et al., 1999), and the brain mask estimated previously was refined with a custom variation of the method to reconcile ANT-derived and FreeSurfer-derived segmentations of the cortical GM of Mindboggle (RRID:SCR_002438; Klein et al., 2017). Volume-based spatial normalization to one standard space (MNI152NLin2009cAsym) was performed through nonlinear registration with antsRegistration (ANTs 2.3.3), using brain-extracted versions of both T1w reference and the T1w template. The following template was selected for spatial normalization: *ICBM 152 Nonlinear Asymmetrical template version 2009c* (RRID:SCR_008796; TemplateFlow ID: MNI152NLin2009cAsym; Fonov et al., 2009).

*Functional data preprocessing.* For each of the 6 BOLD runs per participant (across all tasks), the following preprocessing was performed. First, a reference volume and its skull-stripped version were generated using a custom methodology of *fMRIPrep*. A B0-nonuniformity map (or *fieldmap*) was estimated based on two (or more) echoplanar imaging (EPI) references with opposing phase-encoding directions, with 3dQwarp (AFNI 20160207; Cox and Hyde, 1997). Based on the estimated susceptibility distortion, a corrected EPI reference was calculated for a more accurate coregistration with the anatomical reference. The BOLD reference was then coregistered to the T1w reference using bbregister (FreeSurfer) which implements boundary-based registration (Greve and Fischl, 2009). Coregistration was configured with six degrees of freedom. Head-motion parameters with respect to the BOLD reference (transformation matrices and six corresponding rotation and translation parameters) are estimated before any spatiotemporal filtering using mcflirt (FSL 5.0.9; Jenkinson et al., 2002). BOLD runs were slice-time corrected to 0.708 s (0.5 of slice acquisition range 0–1.42 s) using 3dTshift from AFNI 20160207 (Cox and Hyde, 1997), RRID:SCR_005927. The BOLD time-series (including slice-timing correction when applied) were resampled onto their original, native space by applying a single, composite transform to correct for head-motion and susceptibility distortions. These resampled BOLD time-series will be referred to as *preprocessed BOLD in original space* or just *preprocessed BOLD*. The BOLD time-series were resampled into standard space, generating a *preprocessed BOLD run in MNI152NLin2009cAsym space*. First, a reference volume and its skull-stripped version were generated using a custom methodology of *fMRIPrep*. Several confounding time-series were calculated based on the *preprocessed BOLD*: framewise displacement (FD), DVARS, and three region-wise global signals. FD was computed using two formulations following Power (2014, absolute sum of relative motions) and Jenkinson (2002, relative root mean square displacement between affines). FD and DVARS are calculated for each functional run, both using their implementations in *Nipype* [following the definitions by Power et al. (2014)]. The three global signals are extracted within the CSF, the WM, and the whole-brain masks. Additionally, a set of physiological regressors were extracted to allow for component-based noise correction (CompCor; Behzadi et al., 2007). Principal components are estimated after high-pass filtering the *preprocessed BOLD* time-series (using a discrete cosine filter with 128 s cutoff) for the two *CompCor* variants: temporal (tCompCor) and anatomical (aCompCor). tCompCor components are then calculated from the top 2% variable voxels within the brain mask. For aCompCor, three probabilistic masks (CSF, WM, and combined CSF + WM) are generated in anatomical space. The implementation differs from that of Behzadi et al. (2007) in that instead of eroding the masks by pixels on BOLD space, the aCompCor masks are subtracted a mask of pixels that likely contain a volume fraction of GM. This mask is obtained by dilating a GM mask extracted from the FreeSurfer's *aseg* segmentation, and it ensures components are not extracted from voxels containing a minimal fraction of GM. Finally, these masks are resampled into BOLD space and binarized by thresholding at 0.99 (as in the original implementation). Components are also calculated separately within the WM and CSF masks. For each CompCor decomposition, the *k* components with the largest singular values are retained, such that the retained components' time-series are sufficient to explain 50% of variance across the nuisance mask (CSF, WM, combined, or temporal). The remaining components are dropped from consideration. The head-motion estimates calculated in the correction step were also placed within the corresponding confounds file. The confound time-series derived from head-motion estimates and global signals were expanded with the inclusion of temporal derivatives and quadratic terms for each (Satterthwaite et al., 2013). Frames that exceeded a threshold of 0.5 mm FD or 1.5 standardized DVARS were annotated as motion outliers. All resamplings can be performed with *a single interpolation step* by composing all the pertinent transformations (i.e., head-motion transform matrices, susceptibility distortion correction when available, and coregistrations to anatomical and output spaces). Gridded (volumetric) resamplings were performed using antsApplyTransforms (ANTs), configured with Lanczos interpolation to minimize the smoothing effects of other kernels (Lanczos, 1964). Nongridded (surface) resamplings were performed using mri_vol2surf (FreeSurfer). First, a reference volume and its skull-stripped version were generated using a custom methodology of *fMRIPrep*. A B0 nonuniformity map (or *fieldmap*) was estimated based on a phase-difference map calculated with a dual-echo GRE (gradient-recall echo) sequence, processed with a custom workflow of *SDCFlows* inspired by the epidewarp.fsl script and further improvements in HCP Pipelines (Glasser et al., 2013). The *fieldmap* was then coregistered to the target EPI (EPI) reference run and converted to a displacements field map (amenable to registration tools such as ANTs) with FSL's fugue and other *SDCflows* tools. Based on the estimated susceptibility distortion, a corrected EPI (EPI) reference was calculated for a more accurate coregistration with the anatomical reference. We did not acquire fieldmaps on all participants. For those participants missing a fieldmap, no fieldmap correction was performed within *fMRIPrep*. All other preprocessing steps for these participants were the same as those indicated above.

Many internal operations of *fMRIPrep* use *Nilearn* 0.6.2 (Abraham et al., 2014), RRID:SCR_001362, mostly within the functional processing workflow. For more details of the pipeline, see the section corresponding to workflows in *fMRIPrep*’s documentation.

*Regions of interest.* As preregistered, analyses included four bilateral regions of interest (ROIs), including the striatum, the hippocampus, the dlPFC, and the ventromedial prefrontal cortex (vmPFC). The striatum, hippocampus, and dlPFC ROIs were selected from the corresponding regions in the Harvard–Oxford Subcortical Probabilistic Atlas (HOSPA) thresholded at 25% (Makris et al., 2006). The striatum ROI was created using a combination of left and right caudate and putamen, due to previous research supporting the role of the dorsal striatum in restrictive food choice in AN (Foerde et al., 2015). The hippocampus ROI was created using a combination of the left and right hippocampus, and the dlPFC ROI was created using a combination of the left and right middle frontal gyrus (MFG). The vmPFC ROI used was selected from a meta-analysis of fMRI experiments examining neural correlates of subjective value (Bartra et al., 2013) All ROIs were transformed to the fMRI template space (MNI152NLin2009cAsym) at 1 mm resolution (HOSPA transformed atlas was accessed via Templateflow.org).

#### GLM model setup

*Food choice task.* A generalized linear model (GLM) analysis on the food choice task fMRI data followed a preregistered model and included nine regressors of interest:

i. Onsets for all correct choice trials (i.e., when choice is consistent with initial rating, meaning the chosen item had the higher rating), modeled with a duration equal to the average RT across all valid choice trials and participants
ii. Same onsets and duration as (i) but modulated by |ΔValue| demeaned across these trials within each run for each participant
iii. Same onsets and duration as (i) but modulated by RT demeaned across these trials within each run for each participant
iv–vi. Similar to regressors (i–iii) but for incorrect trials (i.e., when choice is inconsistent with initial rating, meaning the chosen item had the lower rating)
vii. Onsets for all valid trials and same duration as all other regressors, modulated by demeaned average rating across both food items in a pair
viii. Onsets for all valid trials and same duration as all other regressors, modulated by indicator for left/right response
ix. Onset for missed trials, with duration equal to the duration of the stimulus presentation (3 s)

*Perceptual choice task.* GLM analysis on the perceptual choice task fMRI data followed the preregistered model and included eight regressors of interest:

i. Onsets for all correct choice trials (i.e., when the predominant color in the dynamic display was chosen), modeled with a duration equal to the average RT across all valid choice trials and participants
ii. Same onsets and duration as (i) but modulated by |color coherence| demeaned across these trials within each run for each participant
iii. Same onsets and duration as (i) but modulated by RT demeaned across these trials within each run for each participant
iv–vi. Similar to regressors (i–iii) but for incorrect trials
vii. Onsets for all valid trials and same duration as all other regressors, modulated by indicator for left/right response
viii. Onset for missed trials, with duration equal to the duration of the stimulus presentation (3 s)

Both the food choice task and perceptual choice task GLMs included six head-motion parameters (*x*, *y*, *z* translation and rotation), FD, and DVARS as confound regressors. Volumes with FD >0.5 or standard DVARS >1.5 were scrubbed and included as nuisance regressors. These cutoffs deviated from the preregistration, which indicated that excessive motion would be defined as runs with >4 mm head motion or volumes with FD or raw DVARS >0.9, to remain consistent with fMRIPrep's current definition of motion outliers. As was preregistered, runs with >25% of volumes deemed “to-be-scrubbed” were excluded from analyses. Participants with >2 excluded runs were excluded from analyses.

#### GLM model estimation and correction for multiple comparisons

Food and perceptual GLMs were estimated using FMRIB's Software Library's (FSL) FMRI Expert Analysis Tool (FEAT) v. 6.0. For each task, first-level time-series analyses were performed for each run for each participant. The first-level contrast images were then combined across runs per participant using fixed effects. Group-level analyses were conducted using mixed effects via FMRIB's Local Analysis of Mixed Effects (FLAME 1) tool. Consistent with the preregistration, whole-brain group-level maps were corrected to control for multiple comparisons using an uncorrected cluster-forming threshold of *z* = 3.1 and corrected extent threshold of *p* < 0.05.

#### Contrasts of interest

We had three main aims for the fMRI analysis. First, we attempted to replicate a well-established finding in healthy individuals of subjective value coding in the vmPFC (Bartra et al., 2013) and test whether neural coding of food preferences differed in AN. Second, we aimed to test differences in neural coding during deliberation of food versus perceptual decisions in HC and AN. Finally, we tested whether patterns of neural connectivity differed between HC and AN during food choice. To accomplish these aims, we implemented the following three contrasts within the described fMRI GLMs.

*Effect of mean value of food pairs*. A contrast of the effect of differences in mean value rating across items in a pair during the food choice task examined subjective value representations. We conducted whole-brain analyses in both groups to identify regions in the brain that show an effect of mean value (i.e., a correlation between mean value across pairs and BOLD activity during all valid trials of the food choice task [regressor (vii) for food choice task GLM]). Results were compared between groups and tasks to assess the interaction between group and mean value of food pairs on BOLD activity within the whole-brain and within a predefined ROI of the vmPFC.

*Effect of RT.* We next conducted whole-brain analyses in both patients with AN and HC to identify regions in the brain that show an effect of RT (i.e., a correlation between RT and BOLD activity during all valid trials for both tasks [regressors (iii) + (vi) for food choice task and perceptual choice task GLMs]). We then compared brain regions that showed an effect of RT between groups and tasks to assess the interactions between group (patients vs HC), task (perceptual vs food), and RT on BOLD activity within the whole-brain and, as specified in the preregistration, within predefined ROIs of the hippocampus and striatum.

*Psychophysiological interaction (PPI).* A preregistered PPI analysis was conducted to identify brain regions that covaried with activity of the striatum. The striatum ROI was used as the seed in the PPI analysis. The striatum seed was first deconvolved to obtain the neuronal time course and then multiplied with the RT task regressor and reconvolved to create the PPI regressor.

For the perceptual choice task, we included 10 regressors in our GLM:

i–viii. Same regressors included in the perceptual choice task GLM
ix. The raw time course extracted from the seed
x. The PPI regressor with the same onsets as (i)

For the food choice task, we included 11 regressors in our GLM:

i–ix Same regressors included in the food choice task GLM
x. The raw time course extracted from the seed
xi. The PPI regressor with the same onsets as (i)

For each task, we conducted a PPI analysis across the whole-brain and within predefined ROIs of the dlPFC and vmPFC and compared regional effects between groups and tasks.

#### ROI analysis of GLM estimates

Parameter estimates were extracted from predefined ROIs for each task, run, and participant map for the contrasts of interest. The extracted parameter estimates were entered into a mixed-effects linear regression fit in a Bayesian framework using the python package Bambi (Capretto et al., 2022). For analysis of the effect of subjective value on vmPFC BOLD, the model included group (AN or HC) as a regressor of interest. For the analysis of the effect of RT on BOLD, the model included task (food or dots), group (AN or HC), and the interaction between task and group as regressors of interest. For all models, a random intercept and a random slope (when applicable) were entered as random effects per participant. Bambi provided a posterior distribution for all fixed effects. We interpreted an effect as credible if the 94% HDI did not include 0.

#### Bayesian model estimation and Bayes factor computation

To quantify evidence for null group differences (e.g., in cases where frequentist tests yielded nonsignificant results), we computed Bayes factors (BFs) using the Savage–Dickey density ratio method (Wagenmakers et al., 2010). For a given parameter *β* representing a group effect, BF₀₁ is calculated as the posterior density at *β* = 0 divided by the prior density at *β* = 0.

Bayesian mixed-effects models were specified and fit using the python package Bambi (Capretto et al., 2022). Posterior distributions were estimated using No-U-Turn Sampling (NUTS) with four chains and 10,000 draws per chain (DDM and fMRI models) or 2,000 draws per chain (behavioral models). A target acceptance rate of 0.95 was used for all models. Prior predictive distributions were sampled using 40,000 draws (or 10,000 for behavioral models) to evaluate the prior density at the null value.

Priors were specified to be informative but not strongly so, drawing on an independent dataset in which 30 young HC completed similar food choice and perceptual choice tasks used in the present study (Bakkour et al., 2019). This approach grounds the priors in realistic parameter ranges without using any data from the present study. Prior specifications for all models are listed in Table S1.

#### Accounting for clinical variables in analyses

We repeated the analyses described above with the inclusion of clinical variables such as anxiety (measured with STAI), depression (measured with BDI), eating disorder severity (measured with EDE-Q), cognitive dietary restraint (measured with TFEQ-R), and illness duration. Results that account for these additional clinical variables did not differ from those that do not account for them. Thus, we present the results below without these clinical measures accounted for because eight participants (four HC and four AN) were missing at least one of these measures. Results of analyses including these measures are included in the Supplemental Tables.

## Results

We scanned HC (final *n* = 29) and patients with AN (final *n* = 30) while they performed a preference-based task that involved choices between pairs of food items (“food choice task”; Fig. 1*A*,*B*) and a control task that involved choices between two color options based on a visual display (“perceptual choice task”; Fig. 1*C*). We examined differences in behavior (choice and RT), DDM fits, and BOLD activity between the tasks and between groups.

The study was preregistered. For ease of comprehension, we present the results below in narrative form. A comprehensive summary of the preregistered results and unexpected results are presented in Table 1.

**Table 1.** Preregistered hypotheses and corresponding results

Preregistered hypothesis	Result
Behavior on the food choice task will differ between patients with AN and HC	The results (Fig. 2*B*; Table 3; Table S2) do not support this hypothesis
Behavior on the perceptual task will be the same for patients with AN and HC	The results (Fig. 3; Table 4; Table S3) support this hypothesis
Eye tracking will reveal differences in gaze patterns in patients with AN and HC	This hypothesis was not tested due to eye tracking data quality issues
RT will correlate more positively with BOLD activity in the striatum during decisions about food compared with perceptual decisions in patients with AN	The ROI analysis results (Fig. 5*B*) and whole-brain fMRI analysis (Table S8) support this hypothesis. The same effect was also found in the hippocampus (Fig. 5*A*; Table S8). The effect in the hippocampus did not survive whole-brain correction (Table S8)
RT will correlate more positively with BOLD activity in the hippocampus during food-based compared with perceptual decisions in HC	The ROI analysis results (Fig. 5*A*) support this hypothesis, but the effect did not survive whole-brain correction for multiple comparisons (Table S8)
Functional connectivity between striatum and dlPFC will increase with RT during food choice compared with perceptual choice in patients with AN	The results support this hypothesis (Fig. 6; Tables S9)
Functional connectivity between striatum and vmPFC will increase with RT during food choice compared with perceptual choice in HC	The results do not support this hypothesis (Table S9)

### Participants

The final sample consisted of 30 patients with AN (16 restricting, 14 binge-eating/purging subtype) and 29 HC; see Table 2 for participant demographics and clinical characteristics.

**Table 2.** Participant demographic and clinical characteristics

	HC (*n* = 29)		AN (*n* = 28)/atypical AN (*n* = 2)				
	Mean ± SD	Range	Mean ± SD	Range	*t*	df	*p*
Age (years)	25.13 ± 4.85	18–37	24.50 ± 6.52	16–41	0.43	53.54	0.67
BMI (kg/m^2^)	21.75 ± 1.62	19.1–24.5	17.48 ± 1.73	14.5–21	9.70	55.74	<0.001
EDE-Q, Global Score	0.23 ± 0.3	0–1.1	4.42 ± 1.07	1.39–5.9	−20.44	34.74	<0.001
TFEQ-R	4.37 ± 3.43	0–16	18.62 ± 2.82	12–21	−16.90	50.46	<0.001
STAI-T	29.92 ± 7.54	20–51	62.55 ± 9.66	39–80	−13.93	51.53	<0.001
BDI	1.35 ± 2.11	0–7	31.53 ± 9.82	8–47	−16.20	30.58	<0.001
WASI-II FSIQ	114.48 ± 9.12	101–140	107.97 ± 2.48	79–135	2.17	50.91	0.04
Duration of Illness (years)	–		7.37 ± 7.18	0.24–23	–	–	–
Race/ethnicity	*n (%)*		*n (%)*				
African American	3 (10%)		0 (0%)				
Asian	11 (38%)		2 (7%)				
Caucasian	10 (35%)		24 (80%)				
Hispanic	4 (14%)		3 (10%)				
Biracial	1 (3%)		1 (3%)				

BMI, body mass index at time of study; EDE-Q, Eating Disorder Examination Questionnaire (value missing for 4 HC); TFEQ-R, Three-Factor Eating Questionnaire, Restraint Scale (value missing for 2 HC and 1 AN); STAI-T, Spielberger Anxiety Index, Trait version (value missing for 4 HC and 1 AN); BDI, Beck Depression Index (value missing for 1 AN); WASI-II FSIQ, Wechsler Abbreviated Scale of Intelligence, 2nd edition, Full Scale IQ score. Values were compared between groups using Welch's *t* test.

#### Behavioral analyses

**Figure 2.**
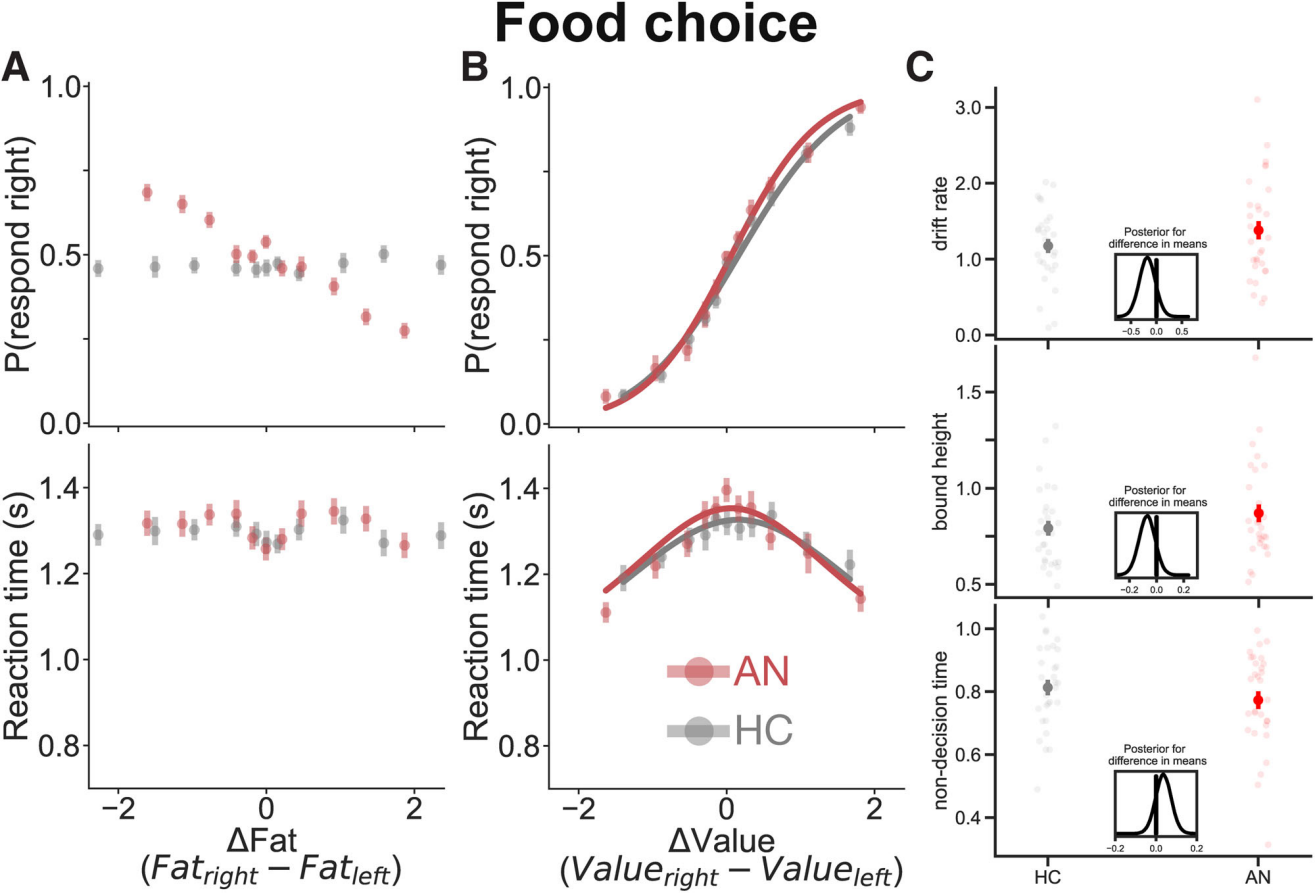
Food choices in AN are more strongly influenced by fat, but decision behavior in both groups is well characterized by the DDM. Choices (top) and RT (bottom) as a function of (***A***) the difference in fat content between choice options and (***B***) the difference in subjective value ratings between choice options. ***C***, HC and patients with AN did not differ in key DDM parameters fit to their choice and RT data. Dark points are means (error bars are standard error of the mean); light dots are individual participant data; colored solid lines are fits of the DDM to the data. The posterior distributions of the BEST (insets) indicate there are no credible differences in means between the two groups.

*Patients with AN choose different foods than HC, but do so using a similar decision-making process*. Patients with AN consistently chose low-fat foods and avoided high-fat foods, a pattern that differed from HC who neither preferred nor avoided fatty foods (Fig. 2*A*). This finding is consistent with the clinical profile and confirms the validity of the food-based decision task in capturing the hallmark maladaptive behavior of AN (see also Hadigan et al., 2000; Mayer et al., 2012; Steinglass et al., 2015; Schebendach et al., 2019; Foerde et al., 2021).

The food choice task design allowed us to account for different preference ratings to probe whether and how the decision-making process unfolds in patients independent of their qualitatively different choices. Overall, we found that the process by which decisions are made—indexed by choice accuracy and model fits—did not differ between the groups (Fig. 2*B*,*C*).

Both HC and patients with AN made choices that were consistent with their initial subjective valuation, as determined by the preference ratings. As expected, patients generally rated preference for foods as lower than controls, but both groups utilized the full rating scale (see Fig. S1*A* for distribution of preference ratings across groups). We examined how the pattern of choices was affected by the difference in value between the two options on each trial (i.e., ΔValue; see Fig. S1*B* for distribution of ΔValue across groups) and found that the consistency in behavioral choices increased as the difference in ratings between the items in a pair increased (main effect of ΔValue in Table 3; Fig. 2*B*, top; Table S3). In other words, both groups were more consistent for “easier” decisions (between options that were more different in value) than for “harder” decisions (between options that were more similar in value; ΔValue × Group BF_01_ = 4.33).

**Table 3.** Regression results for choice and RT during food choice task

	Choice			RT		
	Odds ratio	95% CI	*p*	*β*	95% CI	*p*
Intercept	0.94	0.86–1.02	0.12	1.38	1.33–1.43	**<0.001**
ΔValue	5.83	4.33–7.85	**<0.001**	−0.11	−0.13 – −0.08	**<0.001**
Group [HC]	0.91	0.81–1.03	0.128	−0.05	−0.12–0.03	0.215
Avg Value	0.97	0.91–1.03	0.346	−0.11	−0.12 – −0.09	**<0.001**
ΔValue × Group	0.88	0.58–1.32	0.524	0.04	0.00–0.07	**0.026**
ΔValue × Avg Value	1.17	0.93–1.46	0.178	0	−0.02–0.02	0.971
Group × Avg Value	1.03	0.95–1.12	0.483	0.04	0.02–0.07	**0.002**
ΔValue × Group × Avg Value	0.99	0.74–1.33	0.958	0	−0.03–0.03	0.833
*N*	59			59		
Observations	11,847			11,847		
Marginal *R*^2^	0.357			0.081		
Conditional *R*^2^	0.426			0.214		

For RT regression, ΔValue is entered as absolute value of signed ΔValue. Avg Value is included to account for differences in overall value, but its effect on behavior is not discussed.

All *p* values < 0.05 appear in bold.

Choice difficulty can additionally be quantified by examining RT. RTs for both groups were faster for “easier” than “harder” decisions (i.e., main effect of |ΔValue| on RT; Table 3; Fig. 2*B*, bottom; Table S3), though this relationship was more strongly negative among patients with AN (interaction between |ΔValue| and Group on RT; BF_01_ = 0.59; Table 3). There were however no overall differences between patients with AN and HC on choices or RTs (no main effect of Group; Table 3; Table S3). These findings were replicated in an independent sample of participants (Table S2).

The DDM was fit to choices and RTs from the food choice task for each participant, and ΔValue was entered as a proxy for evidence strength for each trial. This model was selected a priori, as it has been previously applied to characterize underlying decision processes for the types of choices utilized in the current tasks (Gold and Shadlen, 2002; Milosavljevic et al., 2011; Bakkour et al., 2019). In the current sample of participants, the DDM choice function—which is determined by parameters that are constrained by both choices and RT—fit the choice data nearly as well as a dedicated logistic function unconstrained by RT (Fig. S2).

The DDM accounted for patterns of behavior in both groups of participants equally well (Fig. 2*B*, solid lines). Furthermore, there were no differences in model parameters between groups (Fig. 2*C*; Fig. S2; Table S4; BF_01_ = 1.85–2.15). These findings suggest that there were no differences between HC and patients with AN in how they engaged in a decision process about what to eat. Though the input to the decision process (i.e., the subjective values placed on food options) differs, the process by which that input is transformed into a behavioral output appears to be the same.

*Patients with AN do not differ from HC in perceptual decision-making.* The perceptual decision-making task offered an opportunity to assess behavior on a control task that shares many of the same features—trial-by-trial decisions, variation in choice difficulty—but for decisions that are based on external (visual) features rather than internal (food) preferences.

**Figure 3.**
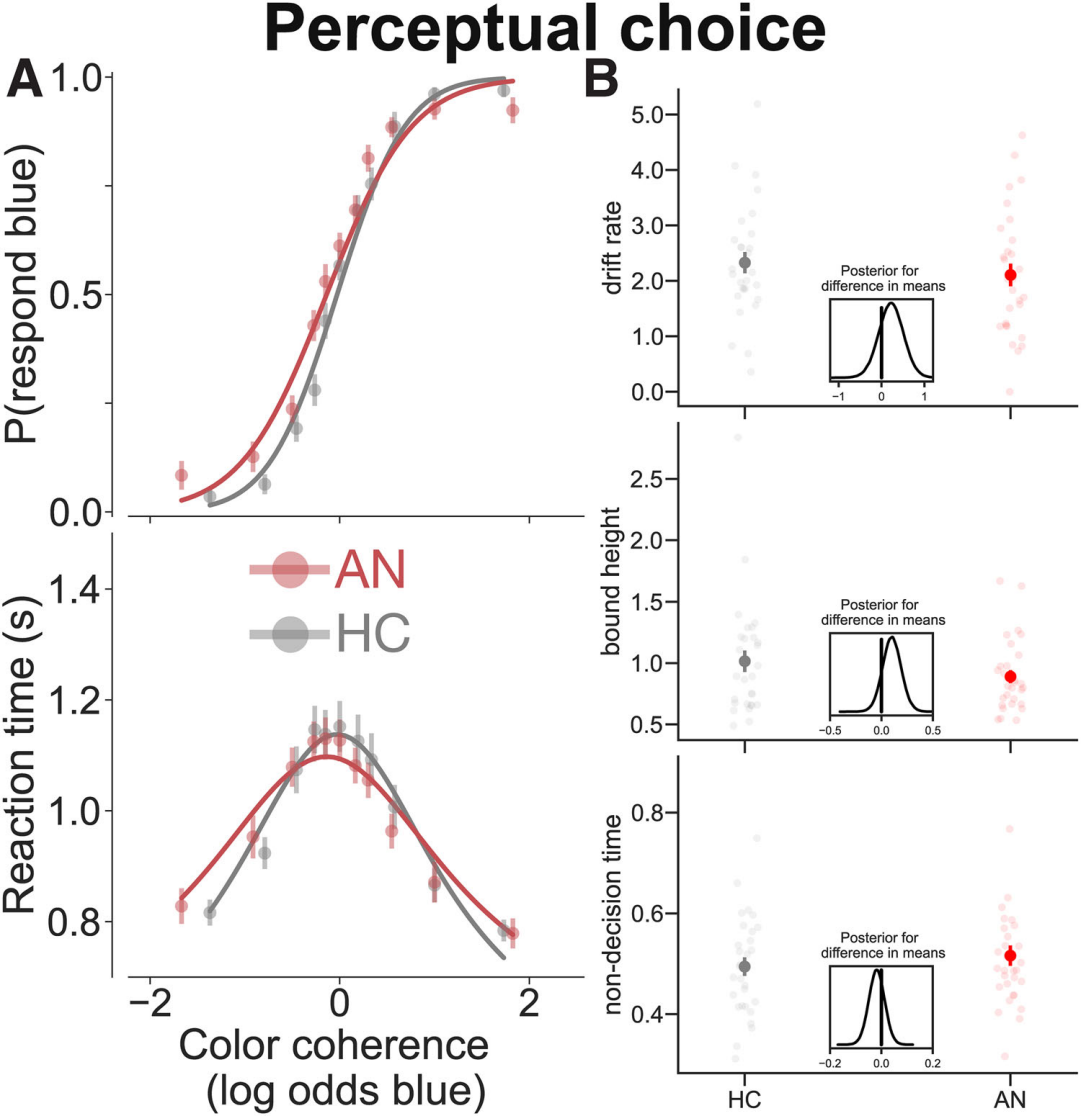
Perceptual decision processes do not differ between patients with AN and HC. ***A***, Choices (top) and RTs (bottom) are plotted against color coherence. Plotted on the top panel is the proportion of trials in which participants responded blue. Plotted on the bottom panel is RT. ***B***, HC and patients with AN did not differ in key DDM parameters fit to their choice and RT data. Data for patients with AN are plotted in red and HC in gray. Points are means (error bars are standard error of the mean); light dots are individual participants; solid lines are fits of the DDM to the data. The posterior distributions of the BEST (insets) indicate there are no credible differences in means between the two groups.

As expected, we found that individuals with AN made similar choices and had similar RTs to HC on the perceptual choice task (Table 4; Table S3). Both groups were more accurate when the display of dynamic dots stimulus contained many more yellow or blue (highly positive or negative values of color coherence; main effect of color coherence in Table 4 and Fig. 3, top). Similarly, RTs for both groups were shorter for decisions on these “easier” trials (Table 4; Fig. 3, bottom). There were no significant group differences on choice or RT, and the effect of color coherence on RT did not differ by group (Color Coherence × Group on RT BF_01_ = 3.33), though there was a marginal interaction between color coherence and group on choice (BF_01_ = 0.95; Table 4). Perceptual task choices and RTs were equally well described by the DDM in HC and those with AN, and there were no group differences in the DDM parameters between groups (Fig. 3*B*; Fig. S2; Table S4; BF_01_ = 1.43–3.20). These findings were consistent with our a priori hypotheses, indicating that patients did not differ from healthy individuals in the behavioral process by which they made decisions based on perceptual features of the external environment. These findings were replicated in an independent sample of participants (Table S2). Moreover, the parallel results between groups confirm that the perceptual task offers a valuable control condition against which to compare brain-related activity in individuals with AN.

**Table 4.** Regression results for choice and RT during perceptual choice task

	Choice			RT		
	Odds ratio	95% CI	*p*	*β*	95% CI	*p*
Intercept	1.66	1.26–2.18	**<0.001**	1.12	1.04–1.20	**<0.001**
Color Coherence	24.77	12.65–48.48	**<0.001**	−0.17	−0.21 – −0.13	**<0.001**
Group [HC]	0.83	0.56–1.22	0.347	0.02	−0.09–0.13	0.744
Color Coherence × Group	2.36	0.90–6.20	0.08	−0.02	−0.07–0.04	0.586
*N*	59			59		
Observations	11,956			11,956		
Marginal *R*^2^	0.622			0.086		
Conditional *R*^2^	0.801			0.308		

For RT regression, Color Coherence is entered as absolute value of signed Color Coherence.

All *p* values < 0.05 appear in bold.

#### fMRI analyses

**Figure 4.**
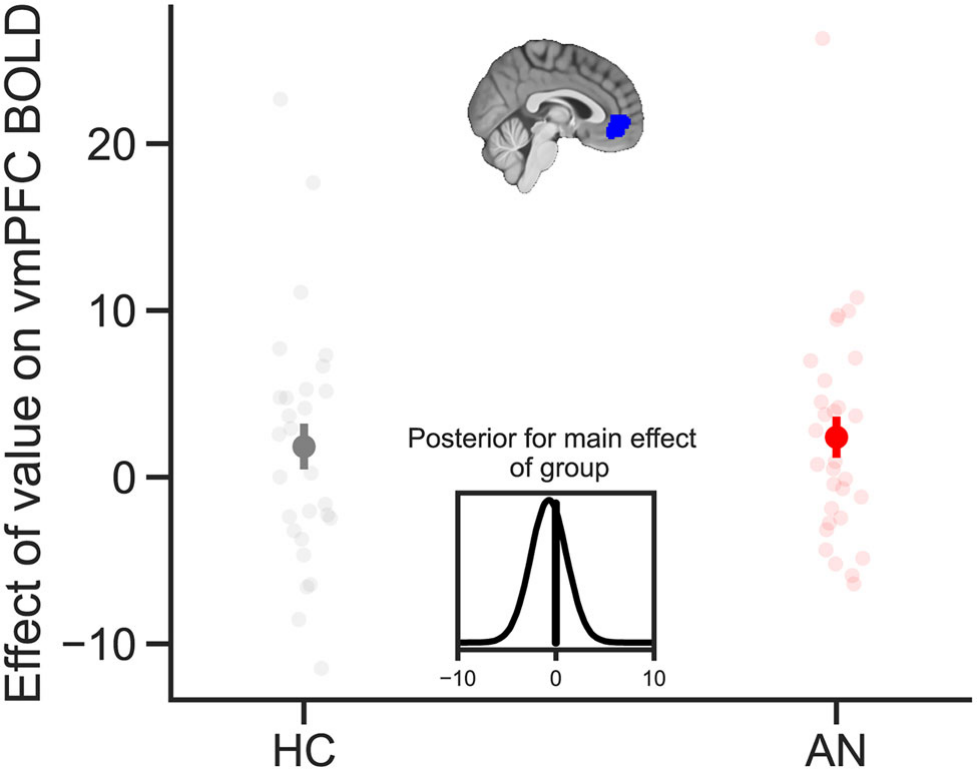
Value-related BOLD activity does not differ between individuals with AN and controls. The average value of the food items presented during each trial of the food choice task correlates with BOLD activity in the vmPFC in patients with AN (red) and weakly in HC (gray). Run-level activations were extracted from the vmPFC ROI shown in blue (top inset). Points are means, and error bars indicate standard error of the mean. Dots are individual participant data. The posterior distribution of the main effect of group in a mixed-effects Bayesian regression (lower inset) indicates there is no credible differences between groups.

*Similar representation of subjective value in the brain for both groups*. We first sought to determine whether there are any differences in brain activity between individuals with AN and controls in BOLD activity related to the subjective value. We ran an analysis with the average value of the food items within each trial as a regressor and extracted participant-level parameter estimates from a predefined mask of the vmPFC, a brain region known to code value. As shown in Figure 4 and Table S5, activity in the vmPFC weakly correlated with subjective value in both healthy individuals and patients with AN (i.e., main effect of mean value on vmPFC BOLD across groups, *M* = 2.39; SD = 1.30; 94% HDI = [−0.05 4.83]), with no difference between groups (i.e., no credible effect of group, *M* = −0.72; SD = 1.86; 94% HDI = [−4.23 2.79]; BF_01_ = 5.89). Whole-brain and small-volume correction analyses that investigate the effect of food value on BOLD provide some additional evidence for the lack of group difference in subjective value coding in the vmPFC (Table S6). This suggests that, while the valuation of specific food items deviates starkly between the groups, once these differences are accounted for by the task, the effect of subjective value on brain activity does not differ between the groups.

*Deliberation of food-related decisions elicit activity in different brain systems among patients with AN and healthy individuals*. We next examined choice-related activity in all participants, comparing food-based to perceptual decisions. The time taken to make decisions was entered as a modulated regressor in fMRI analysis to probe deliberation-related brain activation, and parameter estimates were extracted for each task, run, and participant from a priori bilateral hippocampal and striatal ROIs to compare regional deliberation-related activation between types of decisions and groups in a Bayesian regression.

**Figure 5.**
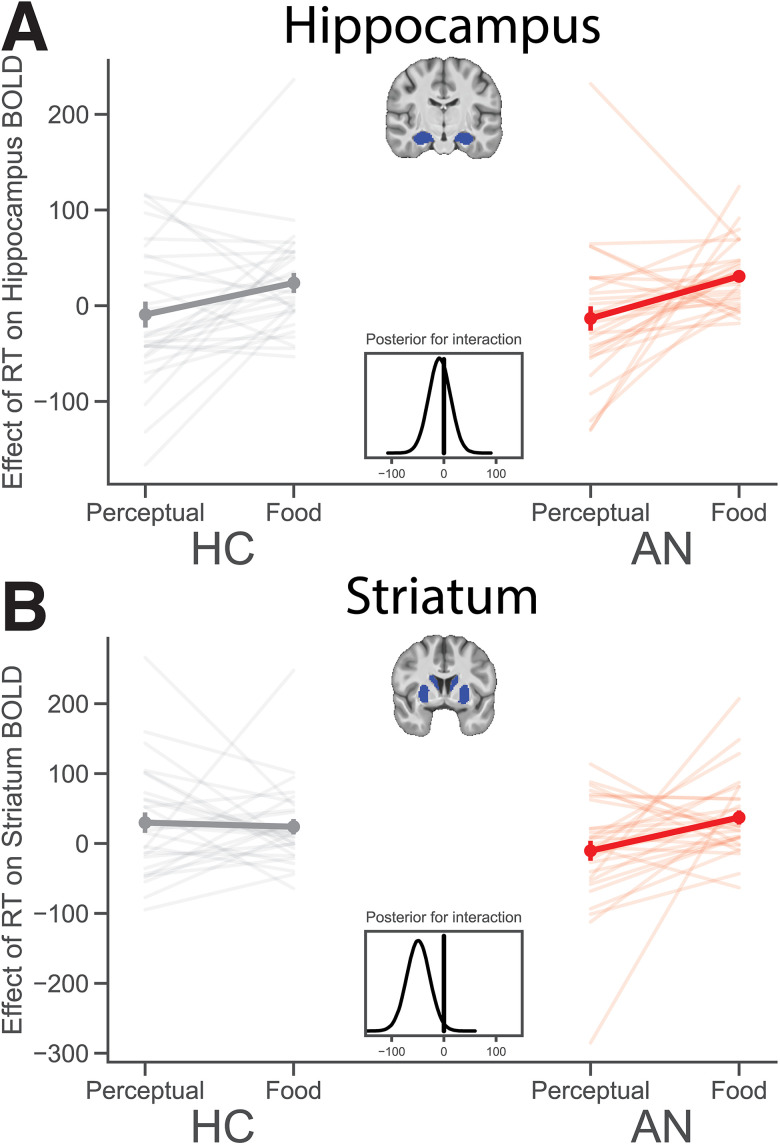
RT-related BOLD activity in the striatum differs between food- and perceptual decisions in AN but not in HC. ***A***, RT correlated with BOLD activity in the hippocampus during food but not perceptual decisions in both HC and AN. ***B***, RT correlated with BOLD activity in the striatum during food but not perceptual decisions in individuals with AN only. Points are means and error bars are standard error of the mean. Light-colored lines are individual participant data. The posterior distribution of the interaction between RT and task on BOLD in a mixed-effects Bayesian regression (lower insets) indicates a credible interaction only in the striatum.

Consistent with prior work (Bakkour et al., 2019), healthy individuals showed greater activation in the hippocampus during deliberation over food compared with perceptual decisions (Fig. 5*A*, left; *M* = 34.91; SD = 16.28; 94% HDI = [4.23 65.39]). The same pattern was evident for patients with AN (Fig. 5*A*, right; *M* = 34.10; SD = 13.41; 94% HDI = [17.73 68.28]), and there was no credible evidence of a difference between groups (i.e., no interaction between task and group on deliberation-related hippocampus BOLD; *M* = −8.25; SD = 20.75; 94% HDI = [−47.40 30.49]; BF_01_ = 4.85; Table S7).

Whole-brain and small-volume correction analyses partially support these ROI results; there is a significant RT-modulated cluster in the hippocampus in AN but not HC, with no difference between groups (Table S8). These findings reinforce the idea that a hippocampus-centered relational memory system supports deliberation over preference-based food choices, not only in healthy individuals but also, unexpectedly, in those with disordered eating.

In contrast, patients with AN differed from healthy individuals in their pattern of deliberation-related activation of the striatum (Fig. 5*B*; Table S8). Indeed, individuals with AN engaged the striatum during deliberation over food choices more than that of perceptual decisions (Fig. 5*B*, right; *M* = 44.89; SD = 15.17; 94% HDI = [16.30 73.52]). Interestingly and consistent with our predictions, the pattern of striatal activation in patients with AN differed from that in healthy individuals (i.e., credible interaction between task and group on deliberation-related striatum BOLD, *M* = −49.24; SD = 22.07; 94% HDI = [−90.67 −7.56]; BF_01_ = 0.44). Whole-brain analyses partially support the ROI findings; there is a significant RT-modulated cluster in the striatum in AN but not HC, with no difference between groups (Table S8). These findings suggest that beyond relying on the hippocampus, as do healthy individuals, patients with AN additionally recruit the striatum during deliberation about what to eat.

*Connectivity between the striatum and dlPFC increases with food choice decision time in patients with AN.* In addition to determining what brain regions were associated with deliberation time for food versus perceptual decisions, we also investigated how the functional connectivity between the striatum and other brain regions got stronger as deliberation time increased, using a PPI analysis. The PPI analysis was used to identify brain regions whose coupling with the striatum was modulated by RT. We hypothesized that, during food-related versus perceptual decisions, HC would show greater connectivity between the striatum and vmPFC during longer when compared with shorter deliberation time. Whereas patients with AN would show greater connectivity between the striatum and dlPFC when RTs are longer when compared with shorter.

**Figure 6.**
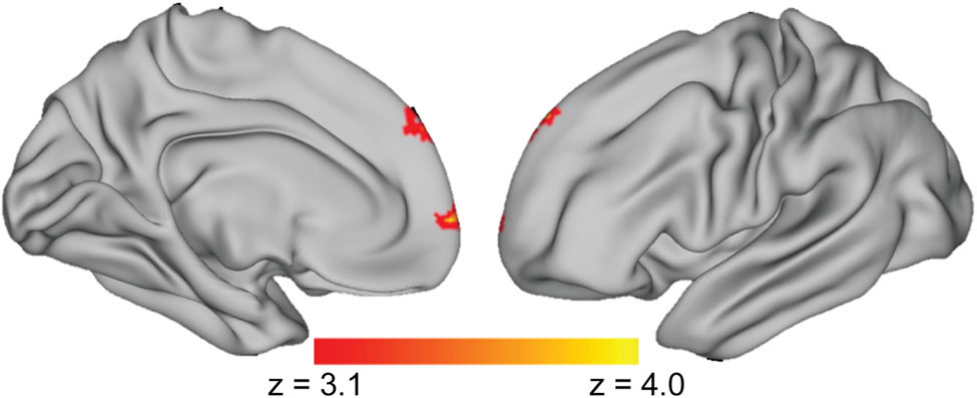
Patients with AN show greater RT-dependent dorsal frontostriatal connectivity during value-based decisions. Medial (left) and lateral (right) view of a semi-inflated surface of the left hemisphere of a template brain, with results of the whole-brain PPI analysis among patients with AN projected onto cortical surface. Compared with the perceptual task data, patients' RT-related activity during the food choice task was more strongly correlated with connectivity between the striatum and the MFG.

Contrary to our predictions, there was not a RT-dependent correlation between activity within the striatum and the vmPFC among HC (Fig. 6; Table S9). Note that this could be due to a lack of deliberation-dependent activation of the striatum among HC (Fig. 5*B*). However, consistent with our predictions, whole-brain and small-volume correction analyses revealed a significant RT-dependent correlation between the striatum and the MFG among patients with AN (Fig. 6; Table S9). These results demonstrate that for patients, longer time to make decisions about food is associated with increased coupling between the striatum and the MFG. There were no three-way interactions between group, task, and RT on striatal–MFG cofluctuations, suggesting that there was no significant group difference in RT-related functional connectivity during food-related versus perceptual decisions.

Together, results of behavioral and imaging analyses indicate that patients make different choices about food than do HC, but there are more similarities than differences in the cognitive computational decision-making process between the groups. After accounting for differences in subjective preferences, the patients show similar consistent use of their own subjective values, and their behavior conforms to a drift-diffusion decision-making mechanism. However, we find that patients differ in the neural mechanism engaged, recruiting more activity in the striatum (in addition to the hippocampus) during deliberation of decisions about food than controls do. Altogether, this suggests that individuals with AN may draw on different sources of internal evidence during choice deliberation of food-based decisions.

*Relationships between decision-model parameters and neural activity within ROIs.* Identifying associations between decision-model parameters and RT-modulated neural activity may help reveal neurobiological correlates of the computational processes supporting food choice. We conducted exploratory analyses testing whether individual differences in fitted decision-model parameters (*B*_0_, *k*, *t*_nd_) were associated with hippocampal or striatal ROI RT-modulated BOLD activity during the food choice task, separately within the HC and AN groups (Table 5). In HC, we observed a negative correlation between *B*_0_ (initial bound height) and hippocampal RT-modulated activity (Spearman *r* = −0.47; *p* = 0.010). In AN, *B*_0_ was negatively associated with RT-modulated activity in both the hippocampus (Spearman *r* = −0.36; *p* = 0.048) and striatum (Spearman *r* = −0.43; *p* = 0.016). None of these associations survived FDR correction for multiple comparisons. Although these analyses were exploratory and likely underpowered, the pattern suggests that higher decision thresholds (larger *B*_0_) may relate to reduced RT–hippocampal coupling in both HC and AN, whereas a comparable *B*_0_-related reduction in RT–striatal coupling may be present only within AN.

**Table 5.** Pearson’s correlations between DDM model parameters (*B*_0_, *k*, and *t*_nd_) and RT-modulated ROI measures (in the hippocampus and striatum) for the food choice task

Group	Parameter	ROI	*r*	*p*	*p* (FDR)
AN	*B*_0_	Hippocampus	−0.36	**0.048**	0.145
		Striatum	−0.43	**0.016**	0.099
	*k*	Hippocampus	0.02	0.914	0.914
		Striatum	0.26	0.163	0.326
	*t*_nd_	Hippocampus	−0.04	0.816	0.914
		Striatum	0.21	0.268	0.402
HC	*B*_0_	Hippocampus	−0.47	**0.010**	0.061
		Striatum	−0.07	0.718	0.851
	*k*	Hippocampus	−0.18	0.360	0.851
		Striatum	0.08	0.664	0.851
	*t*_nd_	Hippocampus	−0.04	0.851	0.851
		Striatum	0.11	0.564	0.851

Correlation values are reported separately for the AN (*n* = 30) and HC (*n* = 29) groups. Values are Pearson's *r* with two-tailed *p* values; *p* (FDR) denotes false discovery rate-corrected *p* values across the set of correlations in the table. No correlations survived FDR correction.

All *p* values < 0.05 appear in bold.

## Discussion

The current study combined measures of decision behavior (RT and choice) with computational models and fMRI data to gain insight into the cognitive and neural processes underlying decision-making in healthy and disordered eating. Importantly, by including both food-related and perceptual decision tasks, we were able to test whether any alterations reflected domain-general differences in decision-making or processes specific to food choice. Contrary to our expectation, we found that individuals with disordered eating (i.e., patients with AN) did not differ from healthy individuals in the cognitive process by which they make decisions in either nonfood- (perceptual) or food-related tasks. Given the striking differences in what patients with AN choose to eat compared with HC, it is surprising that general decision-making processes are preserved in AN. The domain-general findings rule out a broad impairment in evidence accumulation or choice dynamics in AN. Instead, these findings shift the focus of inquiry toward the input to the decision process that leads individuals to choose one food over another (Lloyd et al., 2024)—i.e., the valuation stage.

Despite some differences in food choice RT between groups, the similarities in the way the decision process unfolds in both food and nonfood-related decisions across AN and HC outnumber the differences. In contrast, fMRI results provide some evidence of differences in the neural mechanisms underlying food-related decisions between the groups. Compared with decision time on the perceptual task, results indicate that patients' deliberation time on the food choice task was more related to BOLD activity in the hippocampus and the striatum. Among HC, deliberation-related BOLD activation increased weakly in the hippocampus and not at all in the striatum for food as compared with perceptual decisions, suggesting that patients with AN differ from HC in terms of the brain regions they engage during deliberation of food choice when compared with perceptual decisions. Given prior research showing that the hippocampus contributes to deliberation during food choice in healthy individuals (Bakkour et al., 2019), which we replicate here, it is interesting that patients with AN also show RT-related activation of the hippocampus during food choice when compared with perceptual decisions. In addition to the hippocampus, patients also show greater RT-related activation in the striatum, an effect that is credibly different than in HC. We interpret these fMRI results to suggest that patients with AN recruit different neural systems to compute and generate internal evidence as inputs to the decision process, which leads them to value foods differently and make different choices about food when compared with healthy individuals (Foerde et al., 2015, 2018, 2020; Steinglass et al., 2015). However, why do individuals with AN draw on multiple sources of evidence, while HC appear to draw on only one during food choice?

To help answer this question, we set out to elucidate the brain regions that communicate with those implicated in deliberation during decisions about what to eat. Consistent with our preregistered predictions, functional connectivity analyses reveal stronger coupling between the striatum and MFG (which is encompassed by the dlPFC) during longer deliberations of food choice relative to perceptual choice in patients with AN, but not in HC. Although functional connectivity analyses cannot determine the direction of information flow, we speculate that patients with AN may exert greater control over which neural system or circuit is engaged to compute and generate internal evidence to sample from during deliberation of food choice as the difficulty of the choice increases (i.e., the subjective value of the choice options is closer together, and RT is longer). This interpretation is consistent with the established role of dlPFC in cognitive control processes (Méadhbh and Iris, 2017; Friedman and Robbins, 2022). We proffer that patients with AN might exert more control over the source of evidence they draw on as the difficulty of the decision about what to eat increases, potentially shifting inputs to the decision process from a more flexible cognitive system to one that is more rigid.

Results of the current analyses extend previous research investigating neural mechanisms of food choice in AN by providing evidence to support the idea that maladaptive food choice among patients with AN is driven by differences in inputs to the decision process. An additional novel finding from this study is that, contrary to expectations, maladaptive food choice is not due to irregularities in the cognitive decision-making process itself. Findings of the striatum and hippocampus engagement during deliberation of food relative to perceptual decisions contribute to a growing body of evidence suggesting that dorsal frontostriatal circuitry underlies maladaptive food choice in AN (Foerde et al., 2015, 2020, 2021) and further implicate both the striatum and hippocampus as contributing to decision-making about food among patients.

There are several limitations to the current study that are worth noting. The sample size of 29 HC and 30 patients with AN, while sizable, is smaller than was preregistered and is thus likely underpowered to detect hypothesized whole-brain group differences. Moreover, as both the striatum and hippocampus are known to contribute to many different processes (Anand and Dhikav, 2012; Zhong et al., 2019; Liu et al., 2021), it remains unclear what specific striatal or hippocampal-based processes are relied upon to guide decision-making differentially between groups. Furthermore, the use of a bilateral striatal ROI limits our ability to examine potentially distinct contributions of specific subregions to evidence accumulation during food choice. Future work should examine whether distinct dorsal and/or ventral striatal subregions differentially influence restrictive food choice and value-based decision-making in AN.

Additional limitations relate to the tasks used. The current study relied on binary choice tasks; clinically relevant decisions tend to be multialternative (e.g., deciding what snack to eat from a cabinet with multiple options), and thus, the generalizability of these findings may be limited. Furthermore, because the food and perceptual tasks differ across several dimensions beyond the distinction between internally and externally derived evidence (e.g., reward value, incentive salience), we are unable to determine whether observed effects are food-specific or generalize across value-based decision-making more broadly. The task structure also did not allow us to differentiate between approach and avoidance of food (Melles et al., 2021; Kollei et al., 2022; Lodovico et al., 2023). With regard to generalizability, the current study was conducted among a sample of patients voluntarily receiving highly specialized behavioral treatment for AN; as many patients with AN are not enrolled or engaged in intensive treatment settings, these results may not be representative of all individuals with AN. Future studies may wish to investigate the use of DDMs or other computational models to explain multialternative choice decision-making among a broader pool of individuals with AN.

Finally, while we interpret the current findings to reflect variations in computation of the same information, it is possible that these regions are responsible for encoding distinct information (e.g., different representations or weighting of chosen vs unchosen items, objective nutritional factors, or choice history). Additional investigations are warranted to clarify the nature of the information processed within these regions and to determine whether group differences may arise strictly from variations in informational content or also from differences in neural computations of this content. Direct assessment of these potential differences—for example, by using a representational similarity analysis to probe similarity of patterns of activation across brain regions and groups—could provide a more nuanced understanding of how these mechanisms contribute to food choice behavior within healthy and patient populations.

The current study is the first of its kind to combine tools from computational modeling of behavior and cognitive neuroscience to investigate the cognitive and neural mechanisms that underlie both perceptual and food choice decision-making among patients with AN. Results of this investigation provide initial evidence to suggest that patients follow a behavioral process of evidence accumulation during decision-making that is similar to healthy individuals and extend evidence suggesting these individuals may differ in their recruitment of neural regions during the decision-making process, with increased engagement of the striatum during deliberation of food choice. A critical next step will be to further examine the neural and cognitive processes underlying the valuation stage of decision-making to determine whether, when, and how valuation may change over the course of illness. Whether associations between food choice and striatal activity is a cause or result of the illness also remains unclear and should be the focus of future investigation. Continued investigation of the mechanisms underlying food choice at each stage of the decision-making process may serve to elucidate the substrates of decision-making in both healthy individuals and psychiatric populations and will potentially allow identification of targets for mechanism-based treatments.

## Data Availability

The data supporting the results reported in this article are maintained by the Eating Disorders Research Unit of the NYSPI and are available upon request from the corresponding authors.
